# Supplementary material for: An OpenCV-Based Approach for Automated Cardiac Rhythm Measurement in Zebrafish from Video Datasets
Source: Biomolecules. 2021 Oct 7;11(10):1476. doi: 10.3390/biom11101476 (PMC8533103; doi:10.3390/biom11101476)
Supplement: Supplementary file 1 [file biomolecules-11-01476-s001.zip › biomolecules-1403579-supplementary/File S2_OpenCV SOP.pdf]

# An OpenCV-Based Approach for Automated Cardiac Rhythm Measurement in Zebrafish from Video Datasets

**Ali Farhan**
**PhD fellow**
**Chung Yuan Christian University**
**Supervisor: Prof. Chung-Der Hsiao**

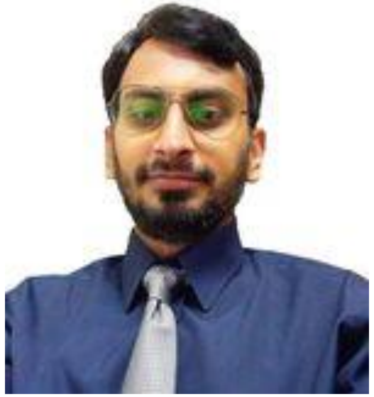

# Introduction

- Using AI-based OpenCV library to detect automatically cardiac rhythm in zebrafish larvae videos

- Advanced features of Python programing language enables the user to perform analysis on 2D video using multiprocessing to select atrium and ventricle region of Interest (ROI) simultaneously.

- Results being validated on the basis of comparison with previously published method of this lab as ImageJ

# Setup for automatic cardiac rhythm analysis for zebrafish larvae

- 2D video recording using high speed CCD
- Installation of Python 3.8 or above version
- Download essential computer vision packages
- Open video in Python (mp4 format)
- Run the program
- Select ROI for atrium and ventricle
- Analysis data
- Results presentation

# Video Recording using HiBestViewer Application (AZ Instrument, Taiwan)

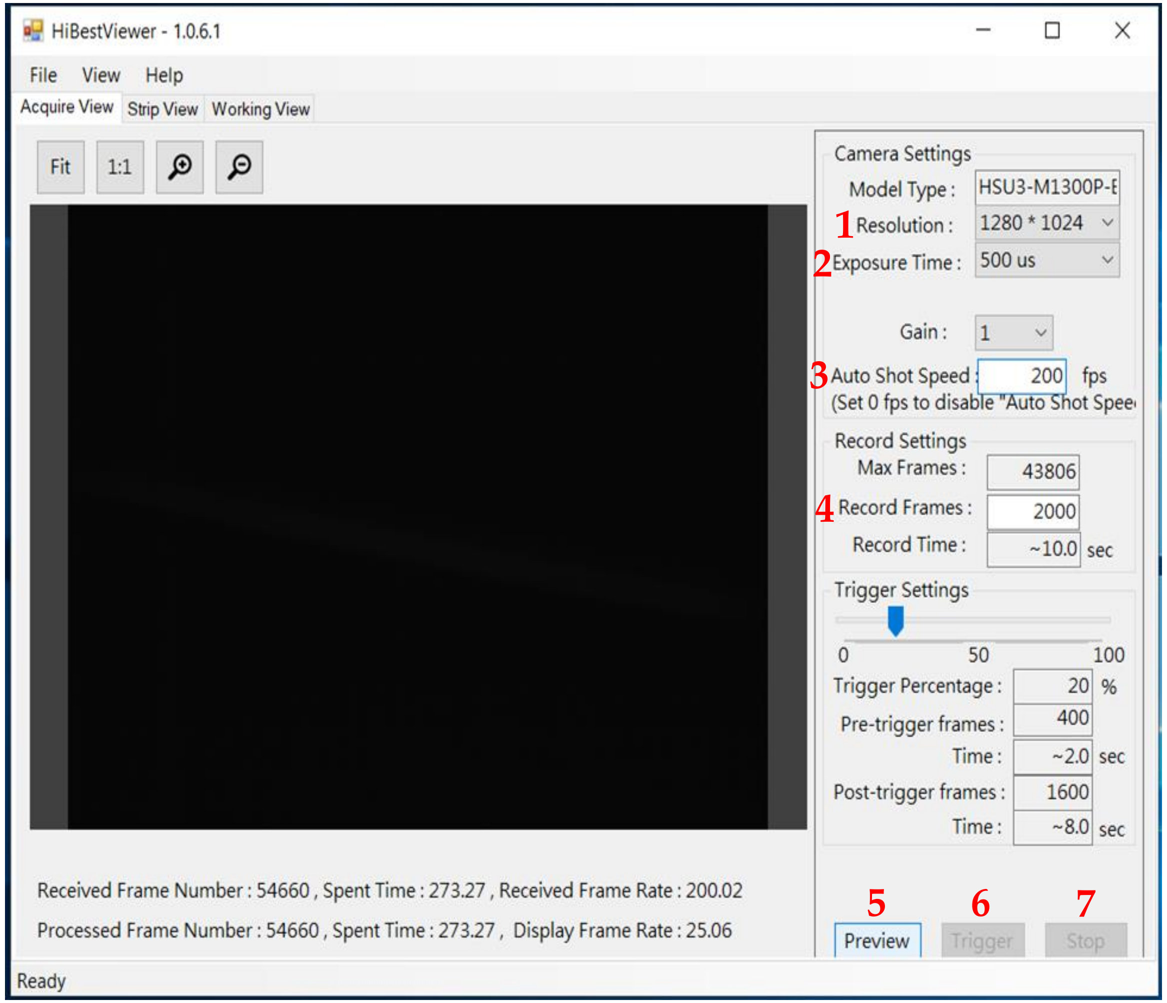


1. **Resolution :** Size of the video taken with a linear correlation to sharpness and clarity.
2. **Exposure time** camera exposed **:** duration of time in which the sensor within the to light
3. **Auto Shot Speed :** number of frames taken in one second for the recording (fps)
4. **Record Frame :** Number of frame set manually by the user to determine the recording length. The final duration of the video is presented in **Record Time**. This number cannot exceed the **Max Frames** or **0**.
5. **Preview :** Start viewing the current condition of the object of interest under the microscope.
6. **Trigger :** Start recording
7. **Stop :** Stop the recording or the viewing.

# Steps to Initialize program

- Put mp4 format video in desired location or folder in system
- Download Python 3.8 version from(https://www.python.org/downloads/)
- Create folder with name as per desired by the user
- Place the zebrafish heart video in the folder and make title as "test.mp4"(at one time one video can be processed)
- Open command prompt window from 'start' panel of system
- Program does not require GPU it can be processed on average CPU
- No need to use anaconda prompt

# Necessary commands use to import libraries in command prompt

- Pip install argparse
- Pip install imutlis
- Pip install opencv-python
- Pip install scipy
- Pip install numpy
- Pip install matplotlib
- Pip install pandas
- Pip install cydets
- Pip install rainflow

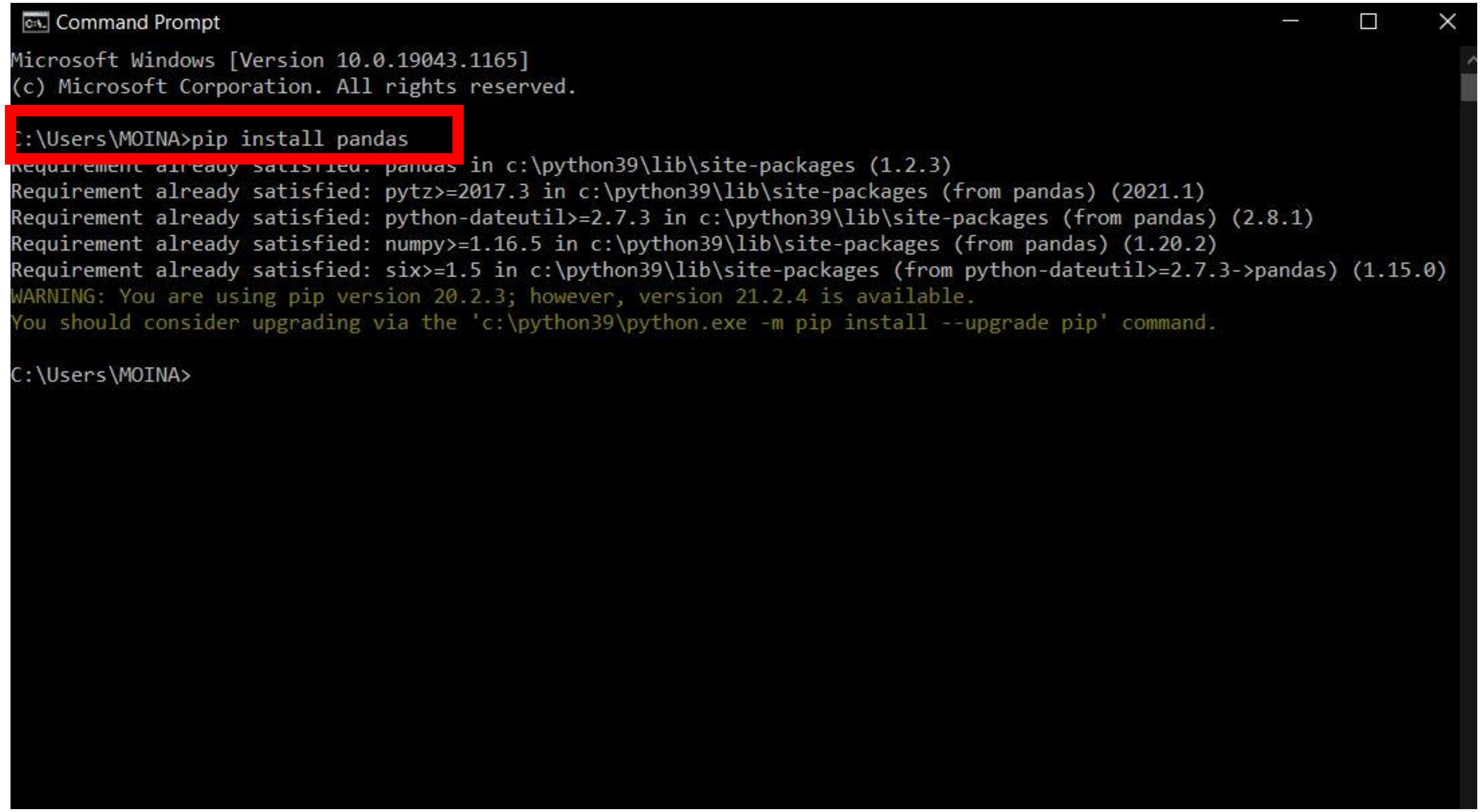

# Operational Flowchart for non-expert users

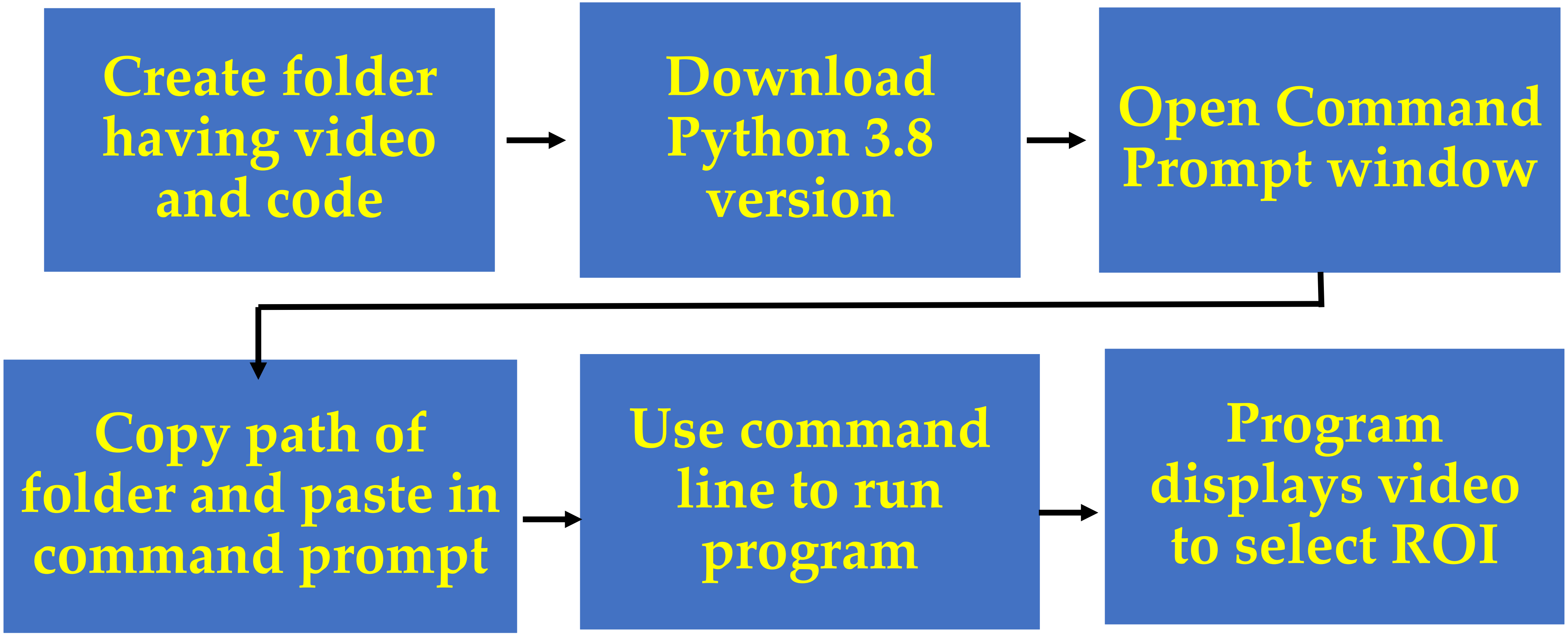

# Folder having code file and video

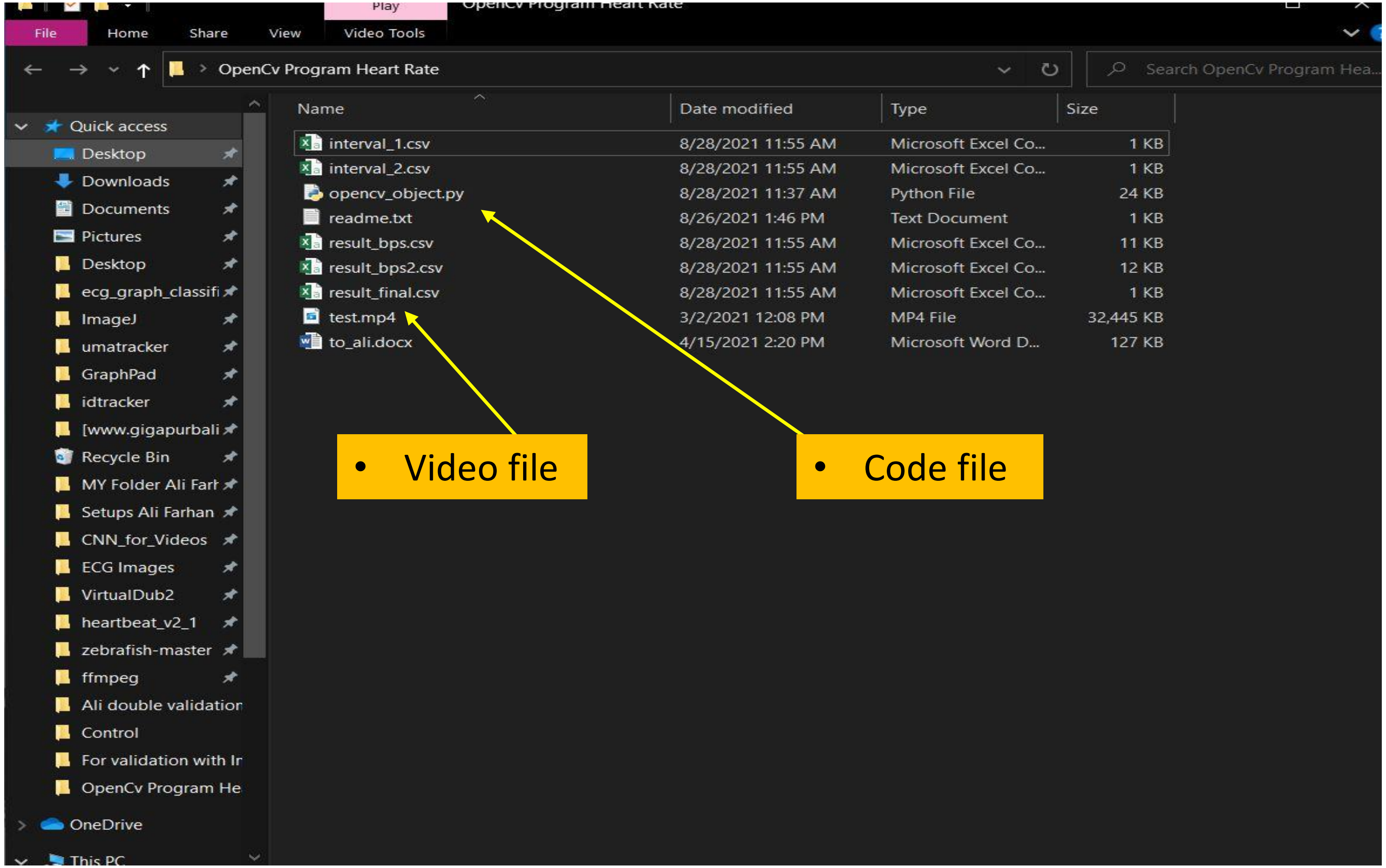

# Command prompt Window

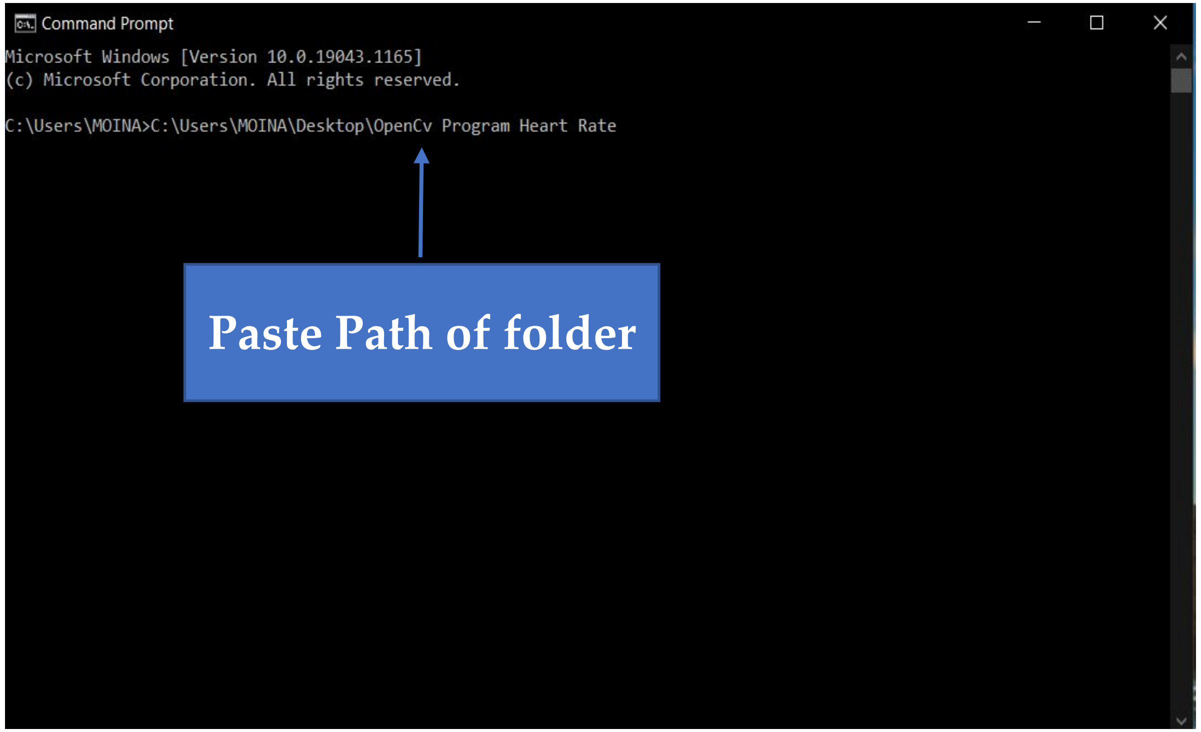


Command Prompt Window

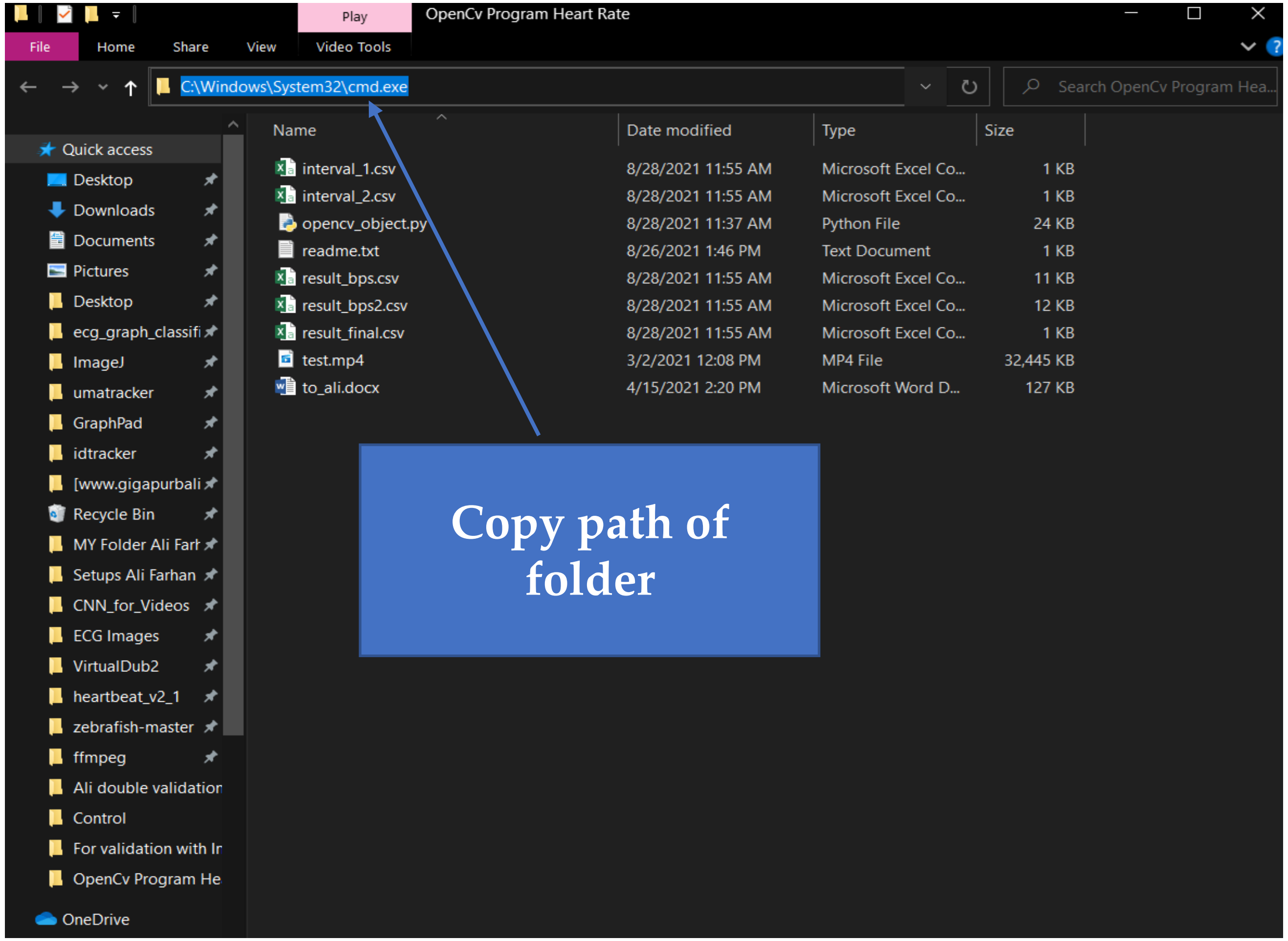

# Continued.....

- By using command line to copy from .txt file in folder paste in the command prompt window

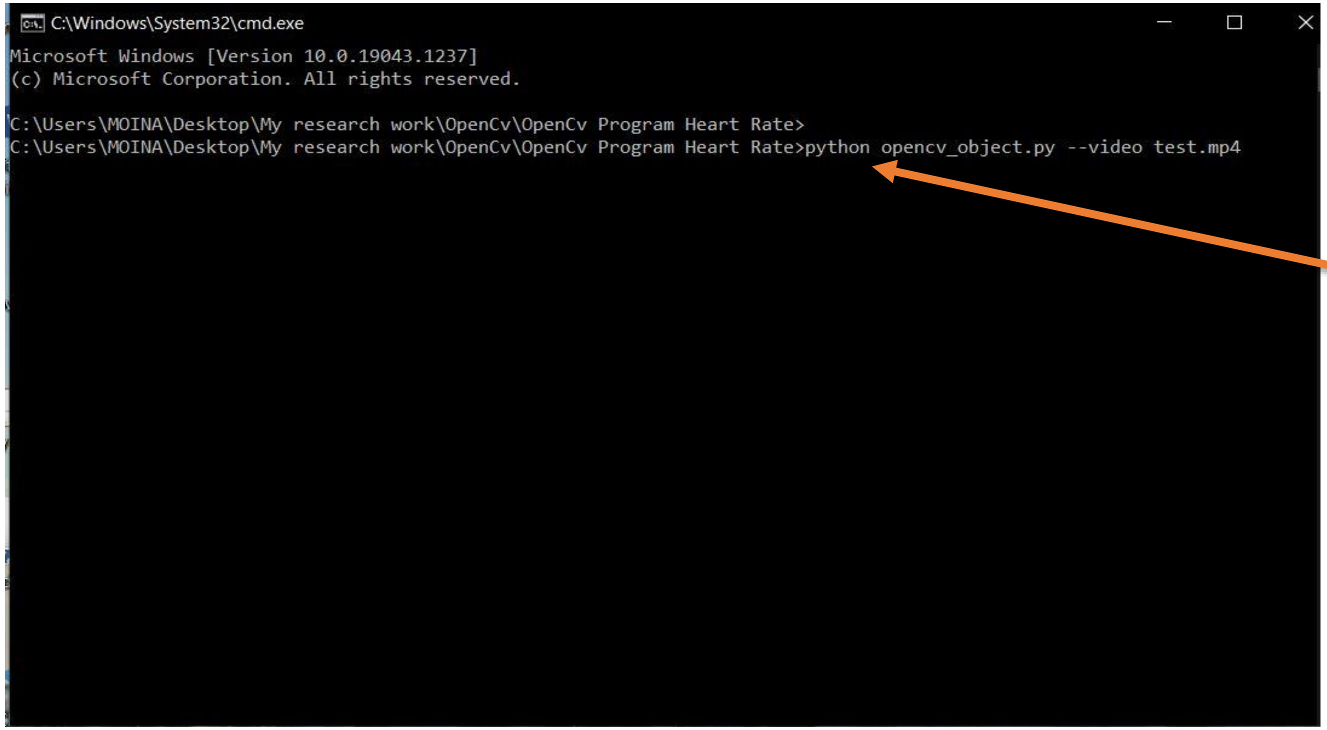


```
# Command to run

python opencv_object.py --video test.mp4
```

# Continued.....

- After completing the installation requirements and program command lines user does not require to do further any step to ‘open the video”. Video shall be opened automatically after copy and paste the command line.
- Once video displayed, users have to select ROI(s) in the video and execution will start automatically till message box displays with ‘heart rate and peaks will be shown as well’.

# Continued…..

- Press Enter to continue
- Program will show message box to select ROI in video file

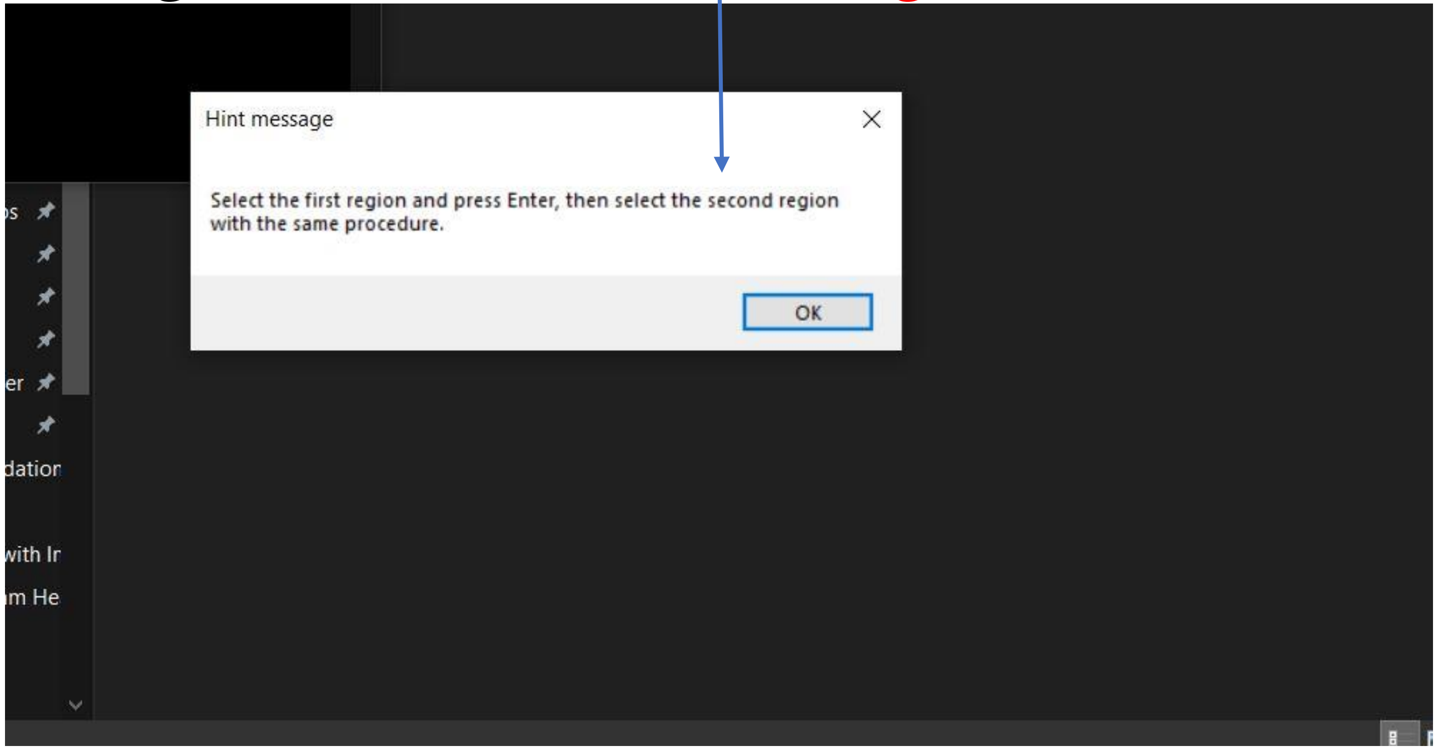

# Selecting ROIs in Zebrafish heart video

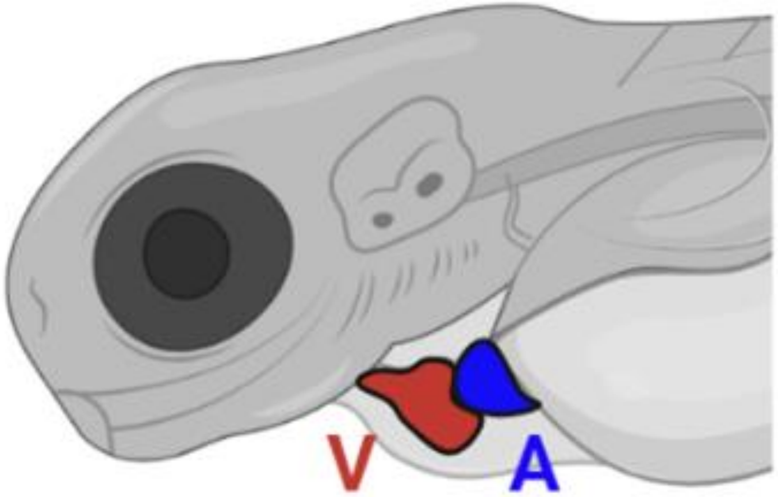


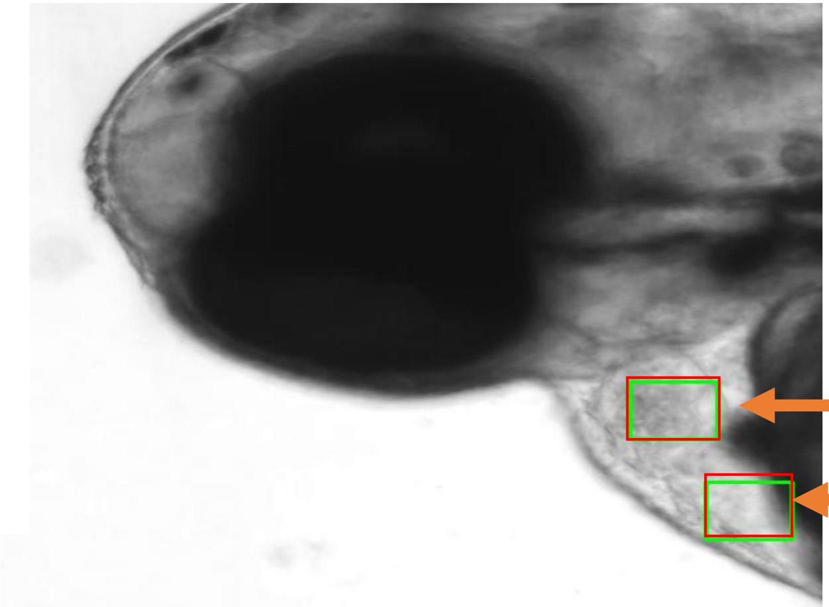

Ventricle (V)
Atrium (A)

It is necessary to select accurate ROI (region of interesting), otherwise results will be compromised as Tool cannot detect atrium and ventricle itself

# Display of Execution

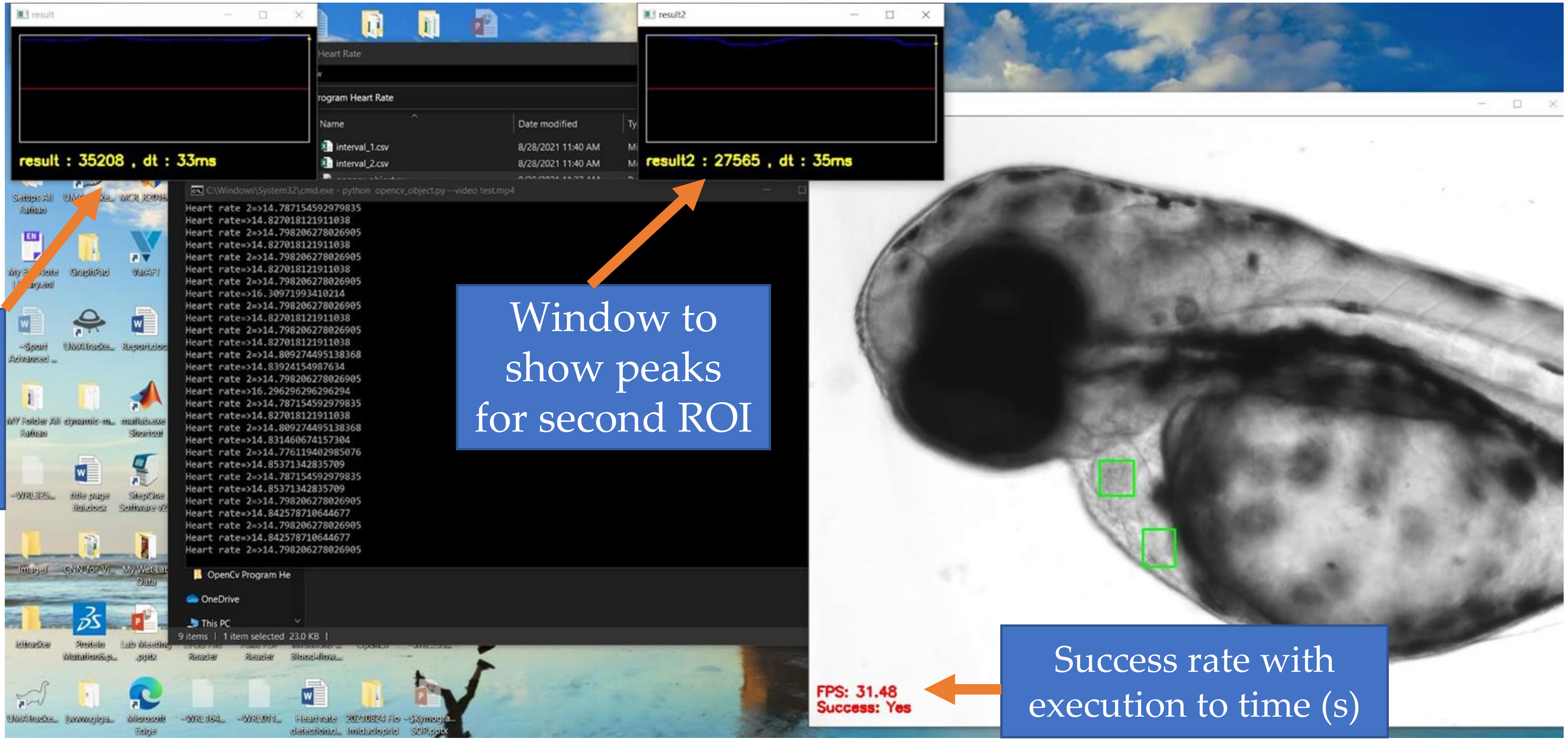


Window to show peaks for first ROI

Window to show peaks for second ROI

Success rate with execution to time (s)

# Calculating Rhythm for different frame rate setting (200 and 60 fps as examples)

```
time_list = [len(s_atri_time), len(s_ventri_time), len(s_atri_heart), len(s_ventri_heart), len(peaks), len(peaks2), len(i
time_len = min(time_list)
s_atri_time = s_atri_time[:time_len]
s_ventri_time = s_ventri_time[:time_len]
s_atri_heart = s_atri_heart[:time_len]
s_ventri_heart = s_ventri_heart[:time_len]
peaks = peaks[1:time_len+1]
peaks2 = peaks2[1:time_len+1]
inv_peaks = inv_peaks[1:time_len+1]
inv_peaks2 = inv_peaks2[1:time_len+1]

final = {
    'Time interval (Atrium)':          s_atri_time,
    'Time interval (Ventricle)':       s_ventri_time,
    'Heart rate (Atrium)':             s_atri_heart,
    'Heart rate (Ventricle)':          s_ventri_heart,
    'High peaks (Atrium)':             peaks,
    'High peaks (Ventricle)':          peaks2,
    'Low peaks (Atrium)':              inv_peaks,
    'Low peaks (Ventricle)':           inv_peaks2
}


df_final = pd.DataFrame(data=final)
df_final.to_csv('result_final.csv', index = False)


get_interval_1(inv_peaks, inv_peaks2, interval_1_cof)
get_interval_2(inv_peaks, inv_peaks2, interval_2_cof)
```

Script first check the length of video then compute inverse peaks

Final data for Time interval for atrium and ventricle in ROIs

# Calculating Rhythm for different frame rate setting (200 and 60 fps as examples)

```
inv_atrium_data = -atrium_data
inv_ventricle_data = -ventricle_data

interval_1_cof = 0.005
interval_2_cof = 200
if real_fps > 35:
    peaks = signal.find_peaks(atrium_data, distance=10)[0]
    peaks2 = signal.find_peaks(ventricle_data, distance=10)[0]
    inv_peaks = signal.find_peaks(inv_atrium_data, distance=10)[0]
    inv_peaks2 = signal.find_peaks(inv_ventricle_data, distance=10)[0]
    interval_1_cof = 0.016
    interval_2_cof = 60
elif 20 < real_fps and real_fps < 35:
    peaks = signal.find_peaks(atrium_data, distance=80)[0]
    peaks2 = signal.find_peaks(ventricle_data, distance=80)[0]
    inv_peaks = signal.find_peaks(inv_atrium_data, distance=80)[0]
    inv_peaks2 = signal.find_peaks(inv_ventricle_data, distance=80)[0]

########################################
# Calculate heartrate and time interval
_, s_atri_time, s_atri_heart = get_heartrate(peaks)
_, s_ventri_time, s_ventri_heart = get_heartrate(peaks2)
```

For 200 fps video, 1/200=0.005 sec

For 60 fps video, 1/60=0.016 sec

# Raw Data Waveform patterns

atrium
ventricle

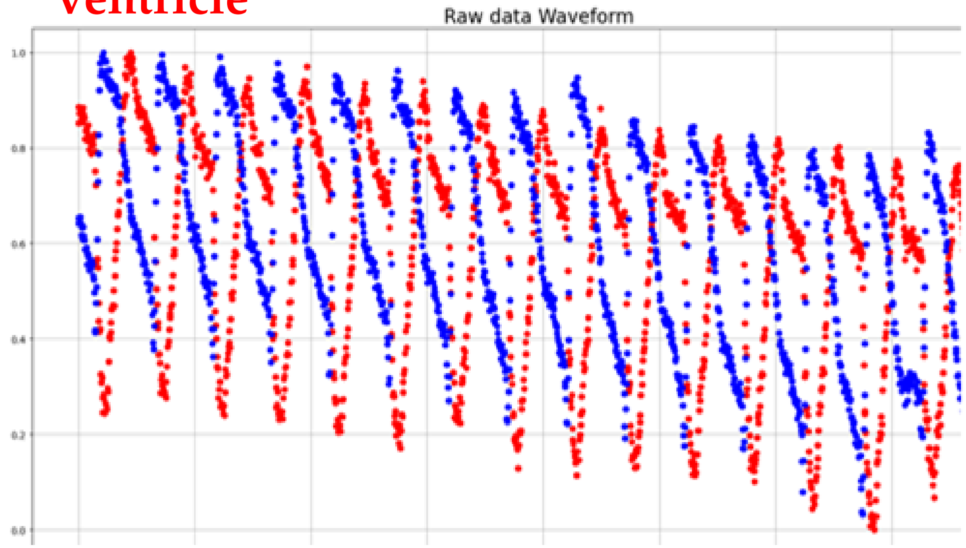


Peaks detected display as raw data (number of frames)

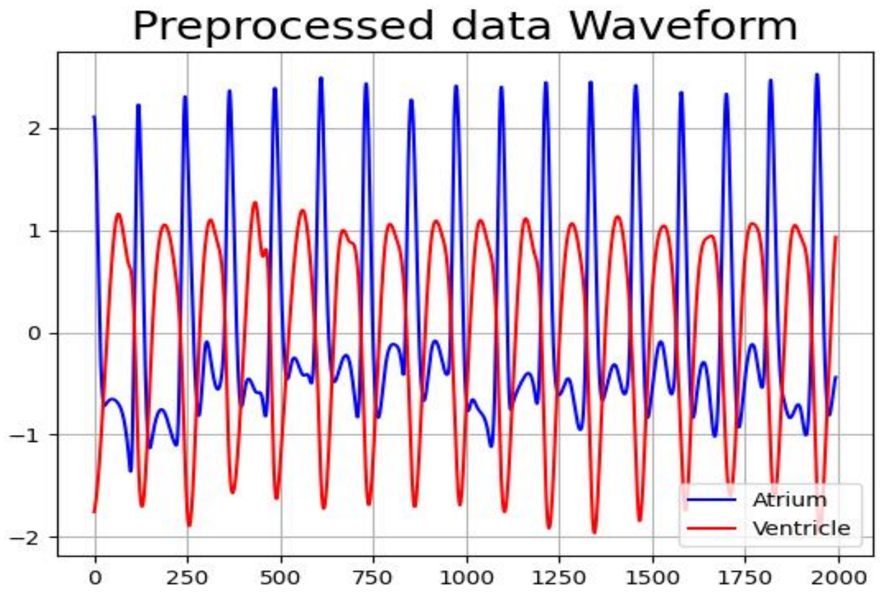


Spline Interpolation used to process the raw data waveform for smoothing

# Smoothing of waveforms

- Using Python scipy-module as interpolate
- Apply multivariate data interpolation
- Griddata (function)
- Check dataset as cubic or linear
- Apply cubic griddata function
- 2D data smoothing with float optional inv
- Call function CloughTocher2DInterpolator

# Functions for smoothening

| | |
|---|---|
| __call__(x, y[, dx, dy, grid]) | Evaluate the spline or its derivatives at given positions. |
| ev(xi, yi[, dx, dy]) | Evaluate the spline at points |
| get_coeffs() | Return spline coefficients. |
| get_knots() | Return a tuple (tx,ty) where tx,ty contain knots positions of the spline with respect to x-, y-variable, respectively. |
| get_residual() | Return weighted sum of squared residuals of the spline approximation: sum ((w[i]*(z[i]-s(x[i],y[i])))**2,axis=0) |
| integral(xa, xb, ya, yb) | Evaluate the integral of the spline over area [xa,xb] x [ya,yb]. |

# Logic design for waveform smoothening

- # ########################################
- # # x_data = Frame,   y_data = Atrium
- # # x_data2 = Frame,  y_data2 = Ventricle
- #   Split time length
- # ########################################
- time_list = [len(s_atri_time), len(s_ventri_time), len(s_atri_heart), len(s_ventri_heart), len(peaks), len(peaks2), len(inv_peaks), len(inv_peaks2)]
- time_len = min(time_list) -1
- s_atri_time = s_atri_time[:time_len]
- s_ventri_time = s_ventri_time[:time_len]
- s_atri_heart = s_atri_heart[:time_len]
- s_ventri_heart = s_ventri_heart[:time_len]
- peaks = peaks[1:time_len+1]
- peaks2 = peaks2[1:time_len+1]
- inv_peaks = inv_peaks[1:time_len+1]
- inv_peaks2 = inv_peaks2[1:time_len+1]

Functions for time interval

Function to extract atrium and ventricle difference (time)

Module in scipy: to find all peaks in array

Atrium=atri
Ventrcile=ventri
Defined variables to save data

# Logic design for waveform smoothening

This function takes a 1-D array and finds all local maxima by simple comparison of neighboring values

- **Parameters**

**points***ndarray of floats, shape (npoints, ndims); or Delaunay* (Data point coordinates, or a precomputed Delaunay triangulation)

**values***ndarray of float or complex, shape (npoints, …)*

Data values as **fill_value***float, optional*

Value used to fill in for requested points outside of the convex hull of the input points. If not provided then the default is nan.

**tol***float, optional*

- Absolute/relative tolerance for gradient estimation.

**maxiter***int, optional*

- Maximum number of iterations in gradient estimation.

**rescale***bool, optional*

- Rescale points to unit cube before performing interpolation.

This is useful if some of the input dimensions have incommensurable units and differ by many orders of magnitude.

Evaluate parameters

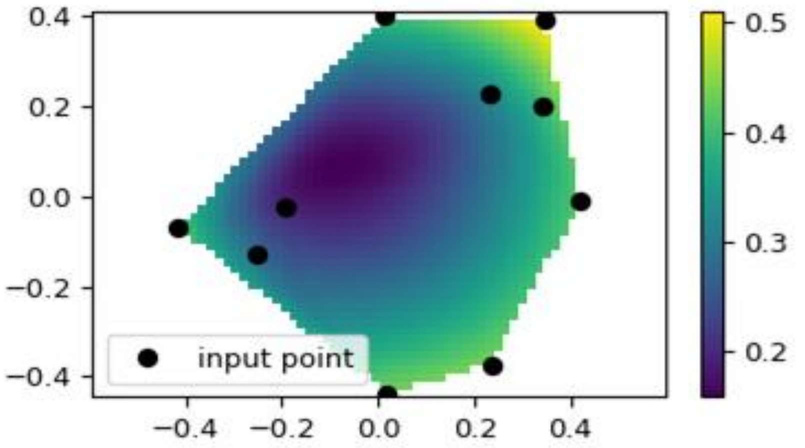

# Logic design for waveform smoothening

Scipy built-in module for edges

# 2-d tests - setup scattered data >>> rng = np.random.default_rng() >>> x = rng.random(100)*4.0-2.0 >>> y = rng.random(100)*4.0-2.0 >>> z = x*np.exp(-x**2-y**2) >>> edges

= np.linspace(-2.0, 2.0, 101) >>> centers = edges[:-1] + np.diff(edges[:2])[0] / 2. >>> XI, YI = np.meshgrid(centers, centers)

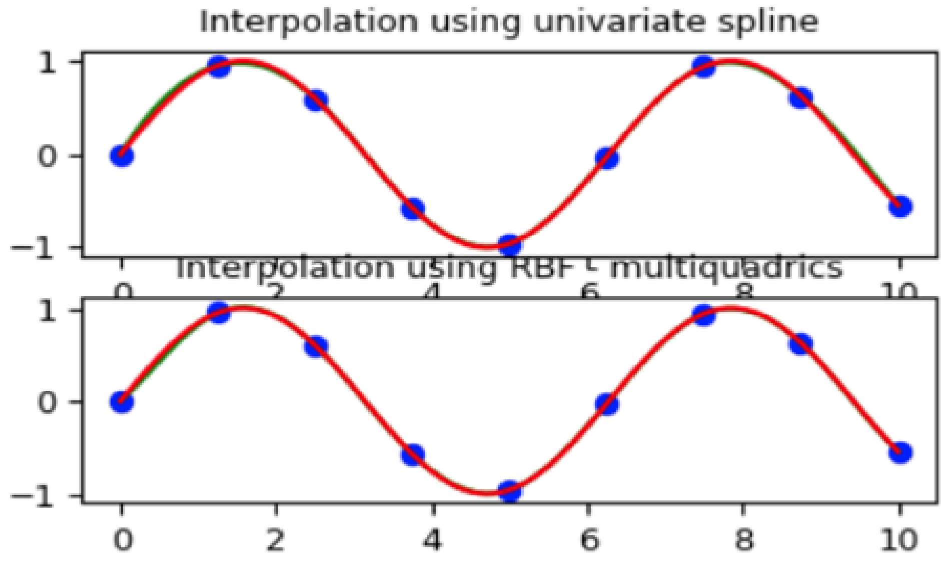


**Reference:**
**https://docs.scipy.org/doc/scipy/reference/tutorial/interpolate.html#d-spline-representation-object-oriented-bivariatespline**

# Finalize dataplots

Use **scipy.interpolate.BivariateSpline** ← Module to confined the x, y peaks with spline interpolation

This describes a spline s(x, y) of degrees kx and ky on the rectangle [xb, xe] * [yb, ye] calculated from a given set of data points (x, y, z).

This class is meant to be subclassed, not instantiated directly. To construct these splines, call either **SmoothBivariateSpline** or **LSQBivariateSpline** or **RectBivariateSpline Compare**

```
import matplotlib.pyplot as plt >>> plt.subplot(221) >>> plt.imshow(func(grid_x, grid_y).T, extent=(0,1,0,1), origin='lower') >>> plt.plot(points[:,0], points[:,1], 'k.', ms=1) >>> plt.title('Original') >>> plt.subplot(222) >>> plt.imshow(grid_z0.T, extent=(0,1,0,1), origin='lower') >>> plt.title('Nearest') >>> plt.subplot(223) >>> plt.imshow(grid_z1.T, extent=(0,1,0,1), origin='lower') >>> plt.title('Linear') >>> plt.subplot(224) >>> plt.imshow(grid_z2.T, extent=(0,1,0,1), origin='lower') >>> plt.title('Cubic') >>> plt.gcf().set_size_inches(6, 6) >>> plt.show()
```

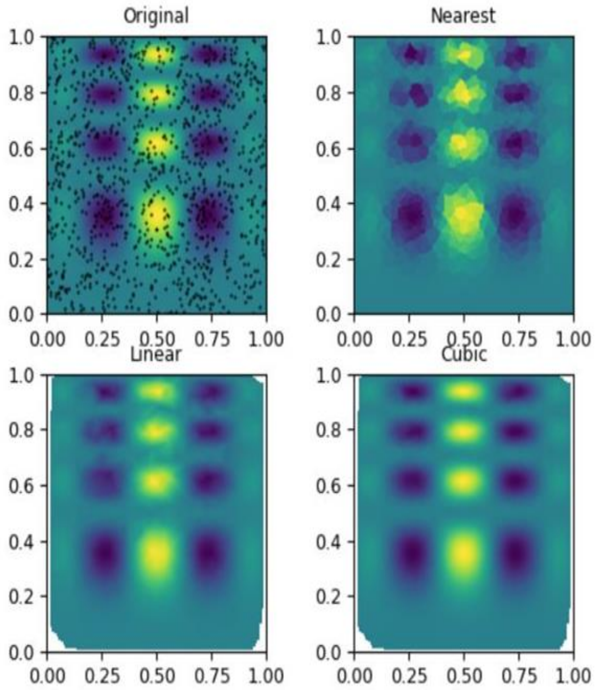

# Finalize dataplots

- s = UnivariateSpline(x_data, y_data, s=50) ← Class for spline fit data 1D
- yy_data = s(x_data)
- peaks = findPeak(yy_data)

- y_data2 = np.array(pplotpix2)
- x_data2 = np.arange(0, len(y_data2))
- y_data2 = normalize_array(y_data2)
- raw_data2 = std_normalize(y_data2)

- s2 = UnivariateSpline(x_data2, y_data2, s=50)
- yy_data2 = s2(x_data2)
- peaks2 = findPeak(yy_data2)

# Script (modified)

# Display Raw data

Function(s) to show title

plt.title("Raw data Waveform", fontsize = 20)

- plt.plot(raw_data, 'bo', label = "Atrium")
- plt.plot(raw_data2, 'ro', label = "Ventricle")
- plt.grid()
- plt.legend(loc = 4)
- plt.show()

Function(s) to plot data and pass grid

Function(s) to show plot

# Display splined data

- plt.title("Preprocessed data Waveform", fontsize = 20)
- plt.plot(yy_data, color = "b", label = "Atrium")
- plt.plot(yy_data2, color = "r", label = "Ventricle")
- plt.grid()
- plt.legend(loc = 4)
- plt.show()

Function(s) to make color as “Blue” for Atrium and Red for “ventricle”

# # Display filtered data

- plt.title("Filtered data Waveform", fontsize = 20)
- plt.plot(atrium_data, color = "b", label = "Atrium")
- plt.plot(peaks, atrium_data[peaks], 'bo', label = "High Peaks of Atrium", alpha = 0.8,)
- plt.plot(inv_peaks, -inv_atrium_data[inv_peaks], 'bo', alpha = 0.5, label = "Low Peaks of Atrium")
- plt.plot(ventricle_data, color = "r", label = "Ventricle")
- plt.plot(peaks2, ventricle_data[peaks2], 'ro', label = "High Peaks of Ventricle", alpha = 0.8,)
- plt.plot(inv_peaks2, -inv_ventricle_data[inv_peaks2], 'ro', alpha = 0.5, label = "Low Peaks of Ventricle")
- plt.grid()
- plt.legend(loc = 4)
- plt.show()

# Smoothing of Peaks shown

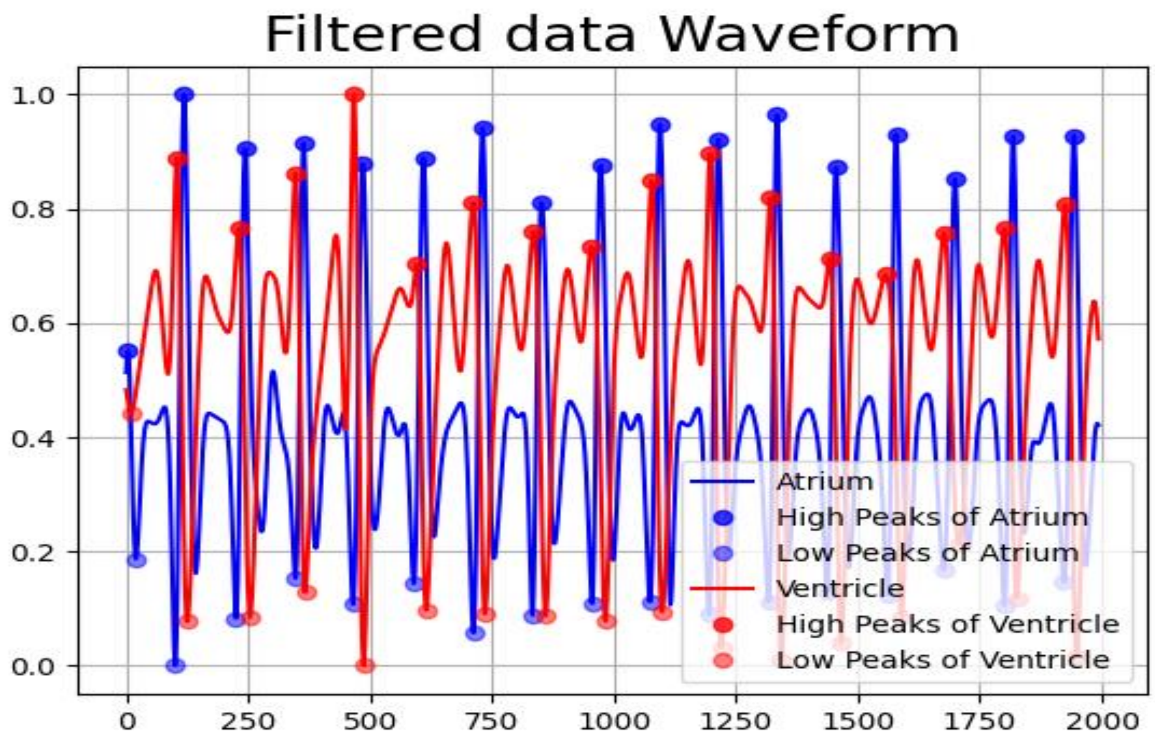


Peaks smoothing performed to get clear visualization as blue for atrium and red for ventricle

# Final Data saved as .csv file

| Name | Date modified | Type | Size |
|---|---|---|---|
| interval_1.csv | 8/28/2021 11:55 AM | Microsoft Excel Co... | 1 KB |
| interval_2.csv | 8/28/2021 11:55 AM | Microsoft Excel Co... | 1 KB |
| opencv_object.py | 8/28/2021 11:37 AM | Python File | 24 KB |
| readme.txt | 8/26/2021 1:46 PM | Text Document | 1 KB |
| result_bps.csv | 8/28/2021 11:55 AM | Microsoft Excel Co... | 11 KB |
| result_bps2.csv | 8/28/2021 11:55 AM | Microsoft Excel Co... | 12 KB |
| result_final.csv | 8/28/2021 11:55 AM | Microsoft Excel Co... | 1 KB |
| test.mp4 | 3/2/2021 12:08 PM | MP4 File | 32,445 KB |
| to_ali.docx | 4/15/2021 2:20 PM | Microsoft Word D... | 127 KB |

| A | B | C | D | E | F | G | H | I |
|---|---|---|---|---|---|---|---|---|
| Time inter | Time inter | Heart rate | Heart rate | High peaks | High peaks | Low peaks | Low peaks | (Ventricle) |
| 116 | 80 | 15.51724 | 22.5 | 121 | 184 | 226 | 124 | |
| 125 | 106 | 14.4 | 16.98113 | 246 | 290 | 347 | 254 | |
| 118 | 171 | 15.25424 | 10.52632 | 364 | 461 | 467 | 376 | |
| 123 | 100 | 14.63415 | 18 | 487 | 561 | 590 | 486 | |
| 123 | 96 | 14.63415 | 18.75 | 610 | 657 | 712 | 617 | |
| 122 | 177 | 14.7541 | 10.16949 | 732 | 834 | 834 | 735 | |
| 121 | 240 | 14.87603 | 7.5 | 853 | 1074 | 955 | 860 | |
| 121 | 123 | 14.87603 | 14.63415 | 974 | 1197 | 1074 | 992 | |
| 121 | 123 | 14.87603 | 14.63415 | 1095 | 1320 | 1196 | 1098 | |
| 121 | 182 | 14.87603 | 9.89011 | 1216 | 1502 | 1318 | 1220 | |
| 120 | 121 | 15 | 14.87603 | 1336 | 1623 | 1438 | 1344 | |
| 120 | 126 | 15 | 14.28571 | 1456 | 1749 | 1561 | 1466 | |
| 123 | 128 | 14.63415 | 14.0625 | 1579 | 1877 | 1676 | 1589 | |
| 121 | 108 | 14.87603 | 16.66667 | 1700 | 1985 | 1801 | 1710 | |

# For AV-VA Interval saved in .csv file as interval 1 and 2 respectively

| | | |
|---|---|---|
| 1 | 0.145 | 0.455 |
| 2 | 0.095 | 0.52 |
| 3 | 0.135 | 0.475 |
| 4 | 0.115 | 0.495 |
| 5 | 0.13 | 0.475 |
| 6 | 0.185 | 0.41 |
| 7 | 0.12 | 0.49 |
| 8 | 0.12 | 0.49 |
| 9 | 0.13 | 0.47 |
| 10 | 0.14 | 0.475 |
| 11 | 0.14 | 0.435 |
| 12 | 0.17 | 0.455 |
| 13 | A_V | V_A |
| 14 | | |
| 15 | | |
| 16 | 0.135769 | 0.47 |

| Name | Date modified | Type | Size |
|---|---|---|---|
| interval_1.csv | 8/28/2021 11:55 AM | Microsoft Excel Co... | 1 KB |
| interval_2.csv | 8/28/2021 11:55 AM | Microsoft Excel Co... | 1 KB |
| opencv_object.py | 8/28/2021 11:37 AM | Python File | 24 KB |
| readme.txt | 8/26/2021 1:46 PM | Text Document | 1 KB |
| result_bps.csv | 8/28/2021 11:55 AM | Microsoft Excel Co... | 11 KB |
| result_bps2.csv | 8/28/2021 11:55 AM | Microsoft Excel Co... | 12 KB |
| result_final.csv | 8/28/2021 11:55 AM | Microsoft Excel Co... | 1 KB |
| test.mp4 | 3/2/2021 12:08 PM | MP4 File | 32,445 KB |
| to_ali.docx | 4/15/2021 2:20 PM | Microsoft Word D... | 127 KB |

com]_DebViC307

# Heart rate measurement for *Daphnia magna*

- In order to determine heart rate of Daphnia as it has single chamber heart the ROI selection was created with bounding box as single ROI selection
- The single chamber heart of Daphnia displays single waveform as compared to zebrafish for two chambers (atrium and ventricle)

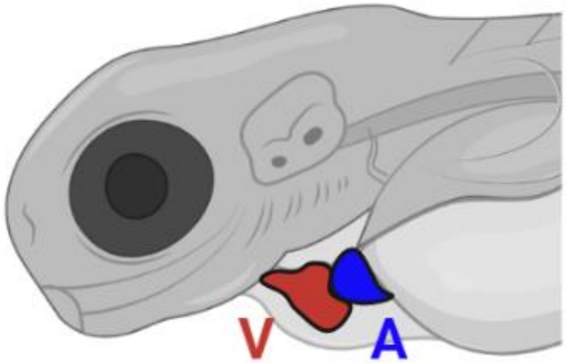


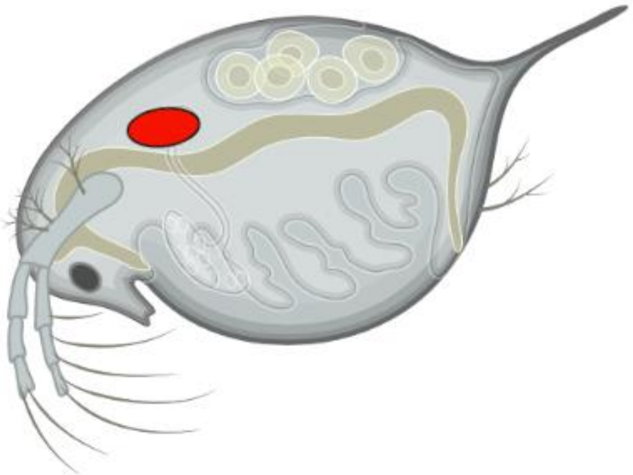

# Heart rate measurement for *Daphnia magna*

- Similar protocol used for *Daphnia magna* heart rate measurement
- User will have to write option "Daphnia/Daphnia_35/Daphnia_IMI" after its shown in command prompt

```
C:\Windows\System32\cmd.exe - python  opencv_object.py --video test.mp4
Microsoft Windows [Version 10.0.19043.1165]
(c) Microsoft Corporation. All rights reserved.

C:\Users\MOINA\Desktop\OpenCv Program Heart Rate>python opencv_object.py --video test.mp4
Input the animal name ( Fish/Daphnia/Daphnia_35/Daphnia_IMI)  Daphnia
```

- Daphnia (For Normal Heart Rate without any toxicity test)
- Daphnia_35 for Temperature effect
- Daphnia_IMI for IMI induced to check Dysregulation

These options created as algorithm designed for zebrafish here we added scales to validate the results of heartbeat in *Daphnia magna* as additional feature

# ROI selection in the heart of *Daphnia magna*

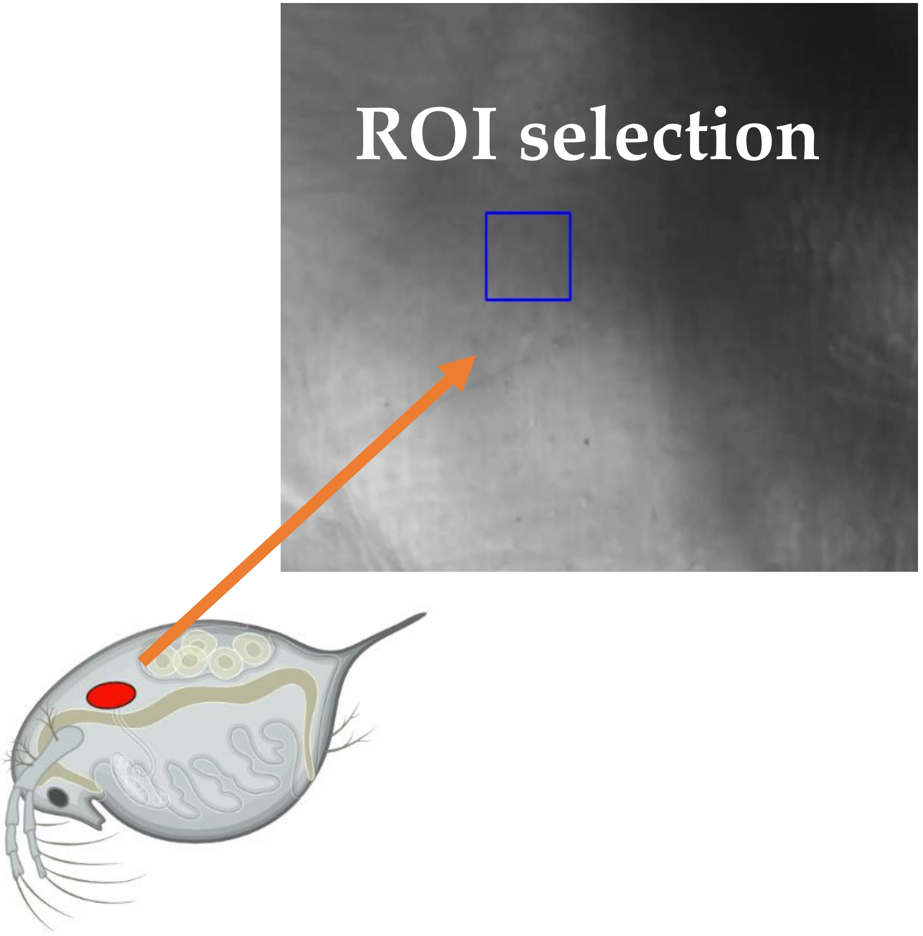


| B | C | D | E |
|---|---|---|---|
| Heart rate | High peaks | Low peaks | (Atrium) |
| 54.54545 | 58 | 39 | |
| 62.06897 | 87 | 69 | |
| 56.25 | 119 | 100 | |
| 62.06897 | 148 | 130 | |
| 56.25 | 180 | 161 | |
| 60 | 210 | 191 | |
| 60 | 240 | 223 | |
| 58.06452 | 271 | 252 | |
| 58.06452 | 302 | 284 | |
| 60 | 332 | 313 | |
| 60 | 362 | 345 | |
| 56.25 | 394 | 374 | |
| 60 | 424 | 407 | |
| 60 | 454 | 436 | |
| 58.06452 | 485 | 467 | |
| 58.06452 | 516 | 497 | |
| 60 | 546 | 528 | |
| 58.06452 | 577 | 558 | |
| 60 | 607 | 619 | |
| 58.06452 | 638 | 651 | |
| 62.06897 | 667 | 680 | |
| 56.25 | 699 | 717 | |
| 60 | 729 | 741 | |
| 58.06452 | 760 | 772 | |
| 60 | 790 | 802 | |
| 60 | 820 | 833 | |
| 60 | 850 | 863 | |
| 54.54545 | 883 | 894 | |
| 64.28571 | 911 | 924 | |
| 58.06452 | 942 | 955 | |
| 60 | 972 | 984 | |
| 58.06452 | 1003 | 1016 | |
| 60 | 1033 | 1045 | |
| 56.25 | 1065 | 1076 | |
| 64.28571 | 1093 | 1107 | |
| 56.25 | 1125 | 1137 | |
| 64.28571 | 1153 | 1167 | |
| 56.25 | 1185 | 1197 | |
| 64.28571 | 1213 | 1227 | |

Data showed more heartbeat events as compared to zebrafish due to *Daphnia magna* has higher heart rate than zebrafish

# Waveform for *Daphnia magna* heartbeat

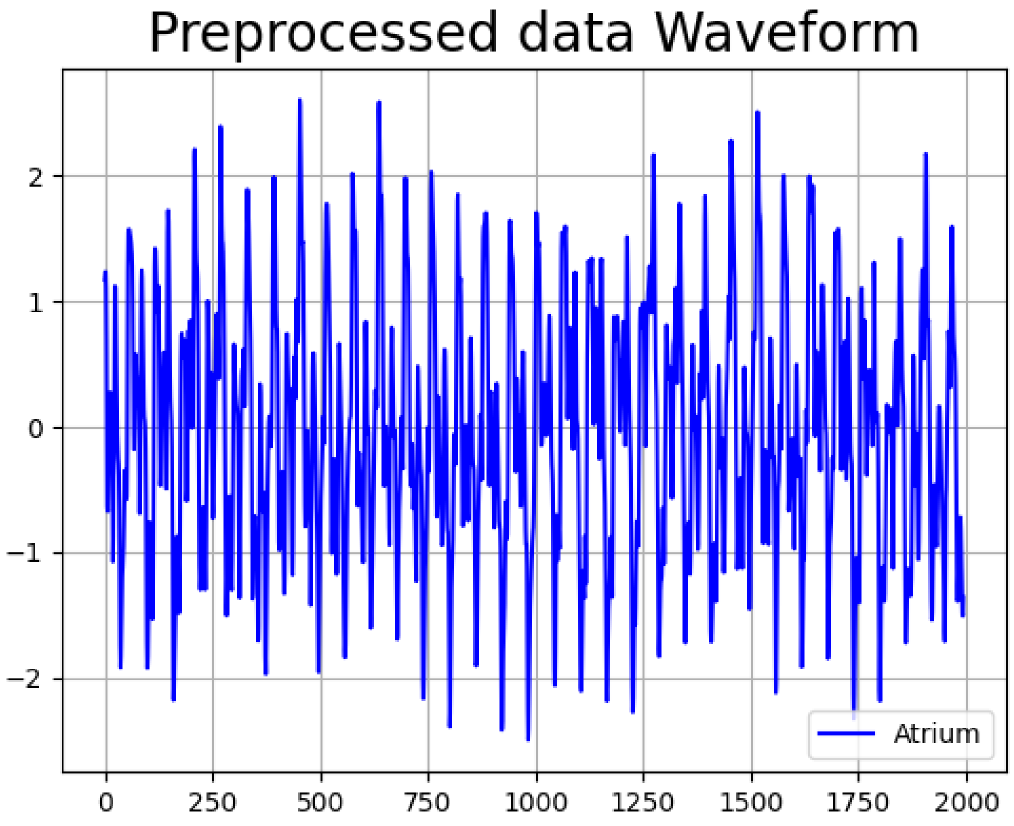


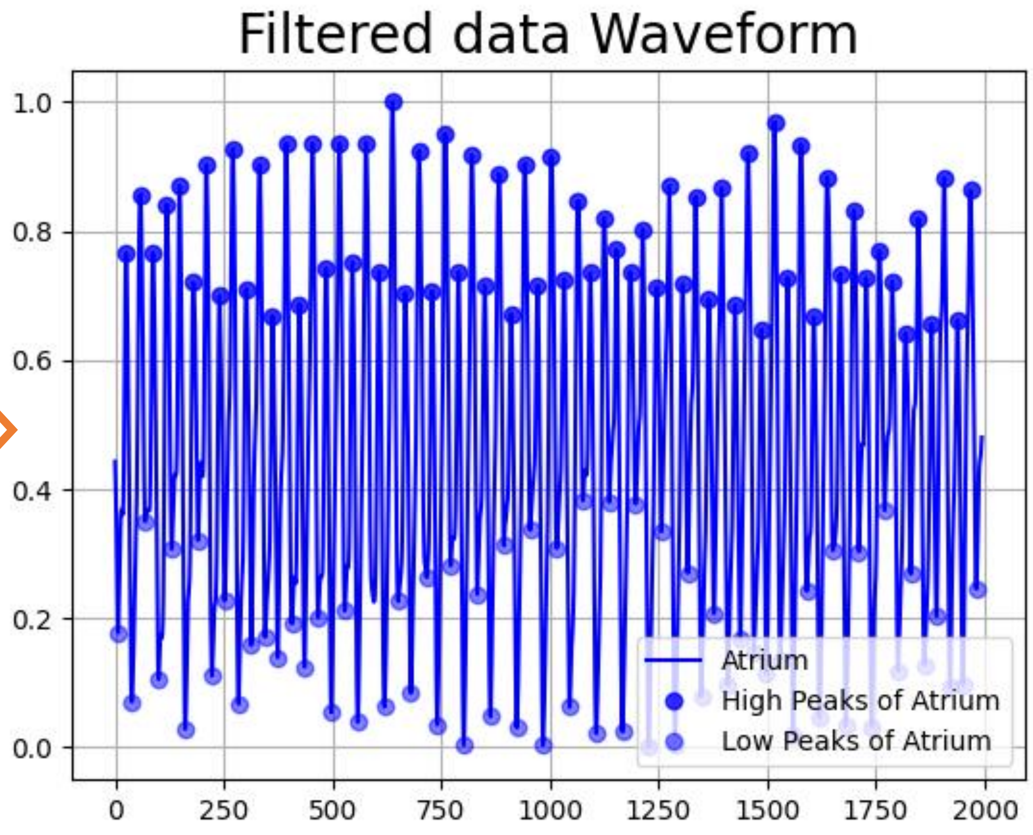


OpenCV method can also be used to detect ultrafast heartbeat in *Daphnia magna*

# Key Features of OpenCV method for heart cardiac rhythm detection

- No need to do video format conversion
- No need any manual calculation for heartbeat
- Automatic detection of rhythm and A-V and V-A intervals
- Results saved in respective folder using csv format automatically
- No need to install or incorporate any external plugins for the analysis
- In zebrafish, both atrium and ventricle rhythm can be detected simultaneously
