# Supplementary figures and images for: An OpenCV-Based Approach for Automated Cardiac Rhythm Measurement in Zebrafish from Video Datasets
Source: Biomolecules. 2021 Oct 7;11(10):1476. doi: 10.3390/biom11101476 (PMC8533103; doi:10.3390/biom11101476)

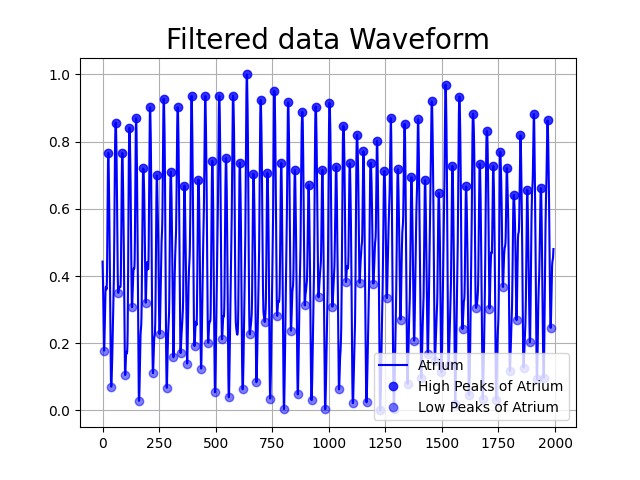

Supplement: Supplementary file 1 [file biomolecules-11-01476-s001.zip › biomolecules-1403579-supplementary/Figure S1.jpg]
